# Folate – a scoping review for Nordic Nutrition Recommendations 2023

**DOI:** 10.29219/fnr.v67.10258

**Published:** 2023-12-26

**Authors:** Anne-Lise Bjørke-Monsen, Per Magne Ueland

**Affiliations:** 1 Department of Medical Biochemistry and Pharmacology, Haukeland University Hospital, Bergen, Norway; 2 Department of Clinical Science, University of Bergen, Bergen, Norway

**Keywords:** folate, folic acid, one-carbon metabolism, homocysteine, nutrition recommendations

## Abstract

Folate is an essential micronutrient for normal development and metabolic function, and folate deficiency is associated with an increased risk of cancer, cardiovascular disease, mental dysfuntion and negative pregnancy outcomes. When estimating folate requirements, one must consider different bioavailability and functionality between synthetic folic acid and dietary folate, together with increased needs of folate in women of fertile age, pregnant and lactating women, preterm and small for gestational age weight infants and individuals who are homozygote for the 5,10-methylenetetrahydrofolate reductase (*MTHFR*) gene polymorphism. In order to achieve an adequate metabolic status based on the metabolic marker total homocysteine, and not merely the absence of clinical signs of folate deficiency, the recommended intake of folate differs according to age, pregnancy and lactation. According to the World Health Organization, a decision limit for folate deficiency in adults is serum folate level below 10 nmol/L, and in women of fertile age a red blood cell folate level below 906 nmol/L in order to prevent neural tube defects. Qualified systematic reviews along with identified relevant literature have been used for this scoping review prepared for the Nordic Nutrition Recommendations 2023.

## Popular scientific summary

- Folate is a micronutrient in the B vitamin complex involved in the synthesis, repair and methylation of DNA.
- Folate is particularly essential for growth and foetal development.
- Foods rich in folate include liver, green vegetables and legumes.
- Folic acid, found in supplements, is the chemically most stable folate form.
- Associations between folate status and chronic diseases, as well as mental function, and pregnancy outcomes, are conflicting.

The aim of this scoping review is to describe the totality of evidence for the role of folate for health-related outcomes as a basis for setting and updating dietary reference values (DRVs) (Box 1). Folate is an essential micronutrient in the B vitamin complex. It is involved in different physiological processes and particularly essential for growth and foetal development because of its role in the synthesis, repair and methylation of DNA, contributing to the formation of new cells and tissues.

*Box 1.* The Nordic Nutrition Recommendations (NNR) 2023.

- This paper is one of many scoping reviews commissioned as part of the Nordic Nutrition Recommendations 2023 (NNR2023) project (19).
- The papers are included in the extended NNR2023 report but, for transparency, these scoping reviews are also published in Food & Nutrition Research.
- The scoping reviews have been peer reviewed by independent experts in the research field according to the standard procedures of the journal.
- The scoping reviews have also been subjected to public consultations (see report to be published by the NNR2023 project).
- The NNR2023 committee has served as the editorial board.
- While these papers are a main fundament, the NNR2023 committee has the sole responsibility for setting dietary reference values in the NNR2023 project.

Folate is present in most foods, and high concentrations are found in liver, green vegetables and legumes. Folate species in food are labile to light and oxidation, and partly destroyed by cooking, compared to the chemically most stable folate form, synthetic folic acid, which is found in supplements (1).

Higher folate requirements are found in infants, children, pregnant and lactating women, patients with intestinal disease, severe skin diseases, haemolytic anaemia, patients taking antiepileptic medications and people with certain gene polymorphisms (2, 3).

The significance of folate status remains conflicting for many conditions, including cancer (4), cardiovascular disease (5), asthma (6, 7), mental function (8, 9) and pregnancy outcomes (10, 11). More than 30 years ago, the British Medical Research Council showed that maternal intake of folic acid starting before pregnancy prevents most cases of infant spina bifida and anencephaly, two major neural tube defects (NTDs) (12). Mandatory food fortification with folic acid is considered a safe and cost-effective intervention to prevent NTDs; however, fortification has not been implemented in many countries, including the Nordic countries (13).

Serum folate is the primary marker of folate status in children, adults and pregnant women. The metabolic marker, plasma total homocysteine (tHcy), increases with decreasing serum folate levels. Plasma tHcy are also affected by vitamin B_12_ and B_6_ status, age and renal function (3). In young children, serum folate concentrations are commonly high (>20 nmol/L) (14). In adults, the World Health Organization (WHO) recommends a serum folate concentration >10 nmol/L and a red blood cell (RBC) folate >906 nmol/L in women of fertile age (15).

In a recent study, median folate intake was 246 µg per day in a Swedish adult population (18–80 years, *n* = 1,797), 25% had a serum folate concentrtion <10 nmol/L, and none of the women of reproductive age had erythrocyte folate concentrations associated with the lowest risk of NTDs (16). Women may reach a preventive RBC folate concentration of more than 906 nmol/L within 4 weeks of supplementation with daily intake of 800 µg folic acid (17), while a dietary folate intake of at least 350 µg per day has been considered necessary to prevent an increase in plasma homocysteine levels of the adult population (18).

## Methods

This scoping review follows the protocol developed within the Nordic Nutrition Recommendations (NNR) 2023 project (19). The sources of evidence used in the scoping review follow the eligibility criteria described previously (20).

The main literature search for this review was performed in MEDLINE on 01 March 2021 with a search string: ((folate[MeSH Terms] AND review[Publication Type] AND (‘2011’[Date – Publication]: ‘3000’[Date – Publication]) AND Humans[Filter])) AND ((‘Diet’ OR ‘Dietary’ OR ‘Food’ OR ‘Nutrition’ OR ‘Nutritional’)). The number of hits was 578. Only one qualified systematic review was identified for folate (21). We also identified relevant literature for this scoping review via ‘snowballing’/citation chasing that was relevant for the background information.

## Physiology

Dietary folates mostly occur as polyglutamyl derivatives and undergo hydrolysis in the gut to monoglutamates before intestinal absorption (22). Folates are transported across the jejunum by a carrier-mediated process, as folic acid, 5-methyltetrahydrofolate (5-MTHF) and 5-formyltetrahydrofolate (23, 24). Folates can also be absorbed by diffusion, a process that is linearly related to luminal folate concentrations and can account for 20–30% of folate absorption at high folate intakes (22).

Folates taken up by the intestinal mucosal cell are reduced to THF, which can either be transferred to the portal circulation without further metabolism or methylated before being transferred. THF is taken up by the liver, methylated to 5-MTHF and 10-formyl-THF, and transported to the peripheral tissues. Folate in the plasma is transported to the tissues as monoglutamate derivatives (2). Within the cell, THF is methylated to 5-methyl-THF, which is converted to folate polyglutamates containing up to seven glutamyl residues. Polyglutamation traps folate inside the cell at concentrations higher than extracellular fluids (25).

The chemically most stable folate form is synthetic folic acid. The bioavailability of food folate is commonly estimated as 50% of folic acid bioavailability when establishing food recommendations, but this should be considered a rough estimate, as data on the bioavailability of food folate vary between 30 and 98% (1, 26, 27). When adults receive daily folate doses <200 µg, little or none is lost in the urine, but at higher doses in the pharmacological range, as used by pregnant women on antiepileptic medication (28), the urinary loss is considerable: 6% of a 1 mg dose, 10% of 2 mg, 50% of 5 mg and 80% of 15 mg (29).

Folate requirements and recommendations for folate intake are expressed as dietary folate equivalents (DFEs) that adjust for the greater degree of absorption of folic acid compared with folate naturally found in foods (1 μg of folate equals 0.6 μg folic acid added to food or taken with food or 0.5 μg folic acid [as a supplement] taken on an empty stomach) (30).

About half of the total body folate pool (5–10 mg) is stored in the liver. Plasma folate consists almost entirely of 5-MTHF (90%) (1). A small fraction of plasma folate is bound to a folate-binding protein.

Folates act as coenzymes for enzymes that mediate single-carbon metabolism. The fully reduced form (tetrahydro-) serves as an acceptor or donor of a single-carbon unit in reactions involved in the synthesis of pyrimidines, purines, serine and methionine (2). Thymidine monophosphate is produced by the methylation of uridine monophosphate. The coenzyme delivering the necessary methyl group in this reaction is 5,10-MTHF, which may be reduced to 5-MTHF for synthesis of methionine (Met) from homocysteine (Hcy) or oxidized to 10-formyltetrahydrofolate for use in purine synthesis (2).

In the Nordic population, 5–8% have a polymorphism in the gene coding for the 5,10-methylenetetrahydrofolate reductase (MTHFR) (C677T, Ala --> Val) (31). This mutation is associated with a decreased activity of the enzyme and results in hyperhomocysteinemia, primarily when folate levels are low. It is recommended that people with MTHFR polymorphism should have a serum folate >15 nmol/L (32).

### Pregnancy

In women of reproductive age, the WHO recommends a RBC folate threshold of <400 ng/mL (906 nmol/L) to be used as an indicator of folate insufficiency, as RBC folate concentrations above this limit will achieve the largest reduction of NTDs (15). Pregnancy is associated with higher demands for folate due to foetal growth and drainage, as well as increased folate catabolism and excretion (33, 34). Serum folate levels decrease continuously during pregnancy and folate stores are depleted after 3 months or sooner if dietary supplements are not provided (35).

### Lactation

During lactation, folate is preferentially taken up by actively secreting mammary glands. 5-Methyltetrahydrofolate is the predominant form of folate in human milk (36). While colostrum is relatively low in folate, milk folate increases during the lactation period (37). Despite reduced maternal folate status, average milk folate levels are reported to be maintained at recommended dietary allowances for infants (38). In a 16 weeks intervention study, there were no differences in total milk folate or in unmetabolized folic acid concentration in the breast milk of women provided with either a low dose of folic acid, a [6*S*]-5-methyl-THF supplement, or a placebo during lactation (39).

### Infants

Maternal folate deficiency is associated with low folate levels in the infants (14, 40). Significantly lower folate levels at birth have also been observed in low-birth-weight (<2,500 g) (41) and premature infants (42). These infants also experience a fall in folate concentrations in early life (43).

In exclusively breastfed infants, plasma folate levels are reported to be elevated after the age of 2 months and are then two- to threefold higher than maternal levels (14, 44, 45). In formula-fed infants, more than 70% have plasma folate concentrations below the lowest concentration for breastfed infants (46). The opposite was observed in Korean infants, where the overall folate intakes in formula-fed infants were significantly higher than those in human milk-fed infants, and this was associated with significantly higher folate and lower tHcy in formula-fed infants than human milk-fed infants at 5 months (47). As the quantity of folic acid in formula milk may differ among countries, this will impact infant folate status.

Median serum folate was 27.0 (IQR= (25th, 75th percentile) 20.4–36.3) nmol/L in Norwegian newborns (4 days) and median 31.6 (IQR= (25th, 75th percentile 21.3–43.3) nmol/L in infants from 6 weeks to 6 months. In this group of infants, exclusive breastfeeding decreased from 73% at 6 weeks to 35% at 6 months (14).

### Older children

**Fig. 1:**
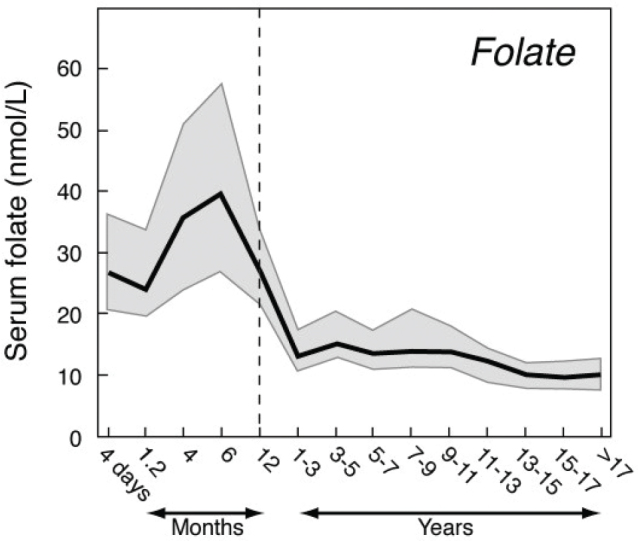
Serum folate concentrations in children aged 4 days to 18 years.

Serum folate remains high up to age 12 months and then decreases to values observed in older children and adults during the first 1–3 years of life (14) (Fig. 1).

#### Malabsorption

Malabsorption occurs with intestinal diseases, such as celiac disease, Crohn’s disease, ulcerative colitis and tropical sprue (3, 48). Folate can bind to food matrices, and many foods (e.g. oranges, lentils, cabbage) contain inhibitors of the intestinal folate conjugase, which reduces folate absorption. Iron and vitamin C deficiencies are associated with impaired food folate utilization (49).

## Assessment of nutrient status

Serum or plasma folate is the primary marker of folate status in children, adults and pregnant women. Intake of food before blood sampling may affect serum folate concentrations; however, most laboratories do not demand a fasting condition. The microbiological assay is considered the gold standard for both serum and RBC folate (50), but more often modern immunoassays are used in clinical laboratories. Although RBC folate is considered to be a better indicator of body stores and nutritional status, there is considerable uncertainty about the reliability of the analytical methods for RBC folate, and many laboratories do not any longer offer this analysis (51,52,53).

A higher intake of folate or folic acid is associated with a higher serum and RBC folate. After initiation of mandatory folic acid fortification in 1998, serum folate centrations more than doubled and RBC folate increased by approximately 50% in the US population (3). Initially, in folate deficiency, serum folate decreases, then plasma tHcy increases and a reduction in RBC folate becomes evident. Folate deficiency gives rise to megaloblastic changes in the bone marrow and other rapidly dividing tissues (54), hypersegmentation in neutrophils and generation of micronuclei in lymphocytes, biomarkers of chromosome breakage or loss (55). Plasma tHcy may increase to 40–50 µmol/L in severe folate deficiency. In patients who are homozygous for the *C677T* polymorphism in the *MTHFR* gene, plasma tHcy may increase to 100 µmol/L. Plasma tHcy also increases with reduced renal function and age, so it is necessary to use age-specific decision limits (56).

Many laboratories still use the 2.5^th^ percentile reference limit to define folate deficiency, ranging from 5 to 7 nmol/L in the Nordic countries. The reference interval is typically defined as the 95% interval between the two reference limits (2.5th and 97.5th percentiles) derived from the distribution of values from an apparently healthy reference population (57). However, a reference interval is merely a description of the folate status in a specific population and will differ according to the diet in the tested population. The mean folate concentration was 29.5 (95% confidence interval: 27.3–31.7) nmol/L in a population-based study including 750 individuals aged ≥12 years in 2017 from Brazil, where folic acid fortification of wheat and maize flours has been mandatory since 2004 (58). In Norway, where folic acid fortification has not been implemented, the mean serum folate was 18.0 (SD 13.8) nmol/L, 39% lower, in 158 Norwegian women of fertile age in 2015 (own unpublished data).

**Fig. 2:**
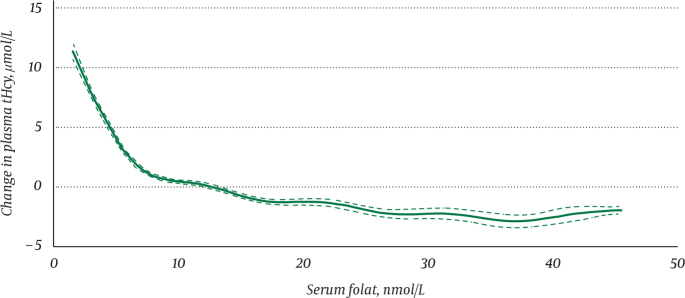
Change in plasma tHcy in relation to serum folate in adults >16 years with normal renal function and serum cobalamin >275 pmol/L (*n* = 12,988) by generalized additive models (GAMs). The values on the y-axis represent the difference from mean plasma tHcy. Published with permission of the Journal of the Norwegian Medical Association (60).

For clinical interpretation of serum folate, one must have clinical decision limits, which defines a value above or below a threshold associated with a significantly higher risk of adverse clinical outcomes or diagnostic for the presence of a specific disease (57). The decision limit may vary according to outcome. When WHO used megaloblastic anaemia as a outcome for folate deficiency, the decision limit was <6.8 nmol/L (59). When WHO used tHcy as a functional marker for folate deficiency, the decision limit was <10 nmol/L (59). The WHO considered that folate status needs to be optimal in women of fertile age to prevent NTDs and suggested that blood cell folate concentrations below 906 nmol/L (serum folate 25–27 nmol/L) should be a decision limit for deficiency in this age group (15). As Figure 2 shows, plasma tHcy starts to increase already when serum folate falls below ~25–27 nmol/L, indicating suboptimal intracellular folate stores, and increases more sharply below ~10 nmol/L, indicating biochemical deficiency (60). A similar relation between serum folate and plasma tHcy is observed in pregnant women (61).

## Dietary intake in Nordic and Baltic countries

Folate is present in most foods, and high amounts are found in liver, green vegetables and legumes. The folates in foods are almost exclusively in reduced form as polyglutamyl derivatives of tetrahydrofolate (FH_4_) (49), and reduced forms are labile to light and oxidation and partly destroyed by cooking. The bioavailability of food folate is commonly estimated to be 50% of folic acid bioavailability when establishing food recommendations, but this should be considered a rough estimate, as data on the bioavailability of food folate vary between 30 (27) and 98% (26), depending on the methodological approach used. Synthetic folic acid, which is a stable oxidized form of pteroylmonoglutamate, is considered to be more bioavailable than natural folate, with a bioavailability when taken with food of 85% and under fasting conditions close to 100% (25, 62).

The average dietary intake in adults in the Nordic countries is 257 µg per day for women and 293 µg per day for men (63). It is notable that average intake in Denmark is approxiately 38% higher in both men and women compared to the other Nordic countries. Median folate intake was 246 μg per day for Swedish adults aged 18–80 years (16).

People with low folate intake, malabsorption or increased folate requirements have a risk of developing folate deficiency. Chronic alcoholism is associated with severe folate deficiency linked to poor dietary intake, intestinal malabsorption, impaired hepatic uptake with reduced storage of folates and increased renal excretion (64). Children and pregnant and lactating women have an increased demand for folate, so have patients with haemolytic anaemia, malignancy, patients undergoing renal dialysis and patients using anticonvulsant drugs (phenytoin, primidone), sulfasalazine (used in the treatment of inflammatory bowel disease), triamterene (a diuretic) or metformin (used in type 2 diabetes) (3).

## Health outcomes relevant for Nordic and Baltic countries

### Cancer

The potential impact of folate on cancer risk has been evaluated with conflicting findings, as various studies have demonstrated increased, risk, no effect and decreased risk. So the relation between folic acid and dietary folate intake, folate status and cancer still remains an unresolved issue (65). Evidence from animal and human studies suggests that folic acid supplementation may prevent neoplastic initiation, but may promote the progression of established precancerous lesions (4).

Elevated tHcy levels and folate deficiency, as determined by serum folate level, were associated with increased overall risk of cancer in a meta-analysis of 83 case-control studies (66). Folate level was inversely associated with most cancer types except prostate, bladder, pancreatic and breast (66). Folic acid supplementation and higher serum levels are associated with increased risk of prostate cancer. Gene polymorphisms may impact cancer risk in certain ethnic groups (67).

The differences in bioavailability and metabolism of synthetic folic acid and natural dietary folate as well as variation in the baseline characteristics of subjects and different methods of folate status assessment in various studies have been suggested as reasons for the controversies regarding colorectal cancer prevention versus promotion (65). Both randomized controlled trials (RCT) and cohort studies have, however, shown beneficial effects of both supplementary folic acid and dietary folate on the primary prevention of colorectal adenomas (68,69,70,71). A recent systematic review including a total of 24 cohort studies involving 37,280 patients and 6,165,894 individuals showed that high folate intake was associated with a reduced risk of colorectal cancer, particularly in people with middle or high alcohol consumption. However, the authors concluded that this still needs to be further confirmed (72).

### Cardiovascular disease

An adequate dietary folate intake (i.e. according to the recommendations) has been inversely associated with both severe and subclinical cardiovascular disease outcomes (73). A systematic review of RCTs from 2016 indicates a 10% lower risk of stroke and a 4% lower risk of overall cardiovascular disease with folic acid supplementation. Folic acid supplementation had no significant effect on risk of coronary heart disease (5). A meta-analysis which included search for both folate and vitamin B_12_ found that homocysteine lowering with B-vitamins among high vascular risk patients who are not taking antiplatelet therapy was related to a significant reduction (29%) in overall stroke risk (74). In the China Stroke Primary Prevention Trial, daily supplementation with 0.8 mg folic acid reduced the incidence of a first stroke by 21%, with greater benefit in those with lower folate levels or higher homocysteine levels (75).

### Mental health

In 2008, a Cochrane review concluded that there is no consistent evidence that folic acid, with or without vitamin B_12_, has a beneficial effect on cognitive function of unselected healthy or cognitively impaired older people (9). However, in Swedish adolescents, higher folate intake and lower homocysteine level have been associated with improved achievement in school, and this effect was consistent after correcting for parental education and other confounders (76). A Norwegian study on 2,189 elderly community subjects followed for 6 years showed higher memory scores in those with higher serum folate (77). A recent meta-analysis found that low folate status was associated with an increased risk of cognitive decline or dementia, whereas folate supplementation protected against the development of dementia (78). After controlling for vitamin B_12_, creatinine, demographic variables and depressive symptom score, a US study from 2005 concluded that RBC folate was directly associated with cognitive function scores and inversely associated with dementia in 1,789 people aged ≥60 years exposed to folic acid fortification (79). A Norwegian study including 2,203 people aged 72–74 years, unexposed to mandatory folic acid fortification, showed that plasma folate was associated with cognitive performance. Among the elderly participants with vitamin B_12_ concentrations in the lower range, the association between plasma folate and cognitive performance was strongest (8).

### Obesity

There is an increasing prevalence of obesity in most parts of the world (80), including the Nordic countries (81). Body mass index (BMI) has been inversely correlated with concentrations of folate in healthy children aged 2 months to 18 years (82), in women of fertile age (83), pregnant women (84) and non-pregnant adults (85).

### Pregnancy outcomes

A Cochrane review reported consistent results showing that folic acid, alone or in combination with vitamins and minerals, prevents NTD, but does not have a clear effect on other birth defects (86) or pregnancy outcomes(10). The US Preventive Services Task Force (87) reviewed systematically results from RCTs on supplementation in pregnancy and NTDs confirming previous conclusions on protective effects. As demands for folate increase during pregnancy, the mother is at risk of developing folate deficiency throughout pregnancy. Folate deficiency has been associated with anaemia and peripheral neuropathy in mothers (88). Recent reviews have found limited evidence for an association between folate status or folic acid supplementation in pregnancy and offspring neurodevelopment (89, 90). However, continued folic acid supplementation beyond early pregnancy, which is currently recommended to prevent NTD, however, is reported to benefit neurocognitive development of the child (91). Use of folic acid supplements during pregnancy was associated with improved neurodevelopment in 4-year-old Spanish children when adjusting for sociodemographic and behavioural factors (92). The absence of folic acid supplementation in early pregnancy was associated with a higher risk of behavioural problems in the offspring at 18 months of age (93). A detrimental effect of high dosages of folic acid supplements (>5,000 vs. 400–1,000 μg/day) during pregnancy on psychomotor development after the first year of life has also been reported (94). A more recent Norwegian study found a 23% increased risk of asthma in children aged 7 years whose mothers had a folate intake of >578 μg per day in pregnancy (7); however, the evidence for an association between folate intake or status in pregnancy and offspring risk of asthma and allergy appears inconclusive (95). There also appears to be inconclusive evidence for an association with protection against hypertensive disorders in pregnancy (96). There is evidence from India of an increased risk of insulin resistance and of obesity in children of women with high serum folate and low serum B_12_ (97). The high serum folate in this population might, however, be secondary to vitamin B_12_ deficiency, which causes an increased amount of functionally inactive methylated folate in the blood.

### Toxicity

The European Food Safety Authority’s (EFSA) upper tolerable intake level of folic acid is set to 200 μg per day for children aged 1–3 years, 300 μg per day for children 4–6 years, 400 μg per day for children 7–10 years, 600 μg per day for children 11–14 years, 800 μg per day for children 15–17 years, 1,000 μg per day for adults >17 years and pregnant and lactating women (98).

Observations indicating adverse effects from excess folic acid intake, elevated serum folate and unmetabolized folic acid concentrations remain inconclusive (99). Although harmful effects in elderly with low vitamin B_12_ status have been reported in several countries, as reviewed recently (100), the data do not yet provide the evidence needed to affect public health recommendations (99).

Adverse effects are exclusively reported from use of the synthetic compound folic acid and no adverse effects have been associated with the consumption of excess folate from foods (101). Only intake of folic acid in excess of 5,000 µg per day may mask haematological manifestations of cobalamin deficiency, as well as antagonize anticonvulsant therapy and affect zinc physiology (4).

Unmetabolized folic acid is detected in nearly all serum samples from US children, adolescents and adults (102). Concerns have been raised about the potentially untoward effects of unmetabolized synthetic folic acid with regard to cancer, depression and cognitive impairment (103). In postmenopausal women unmetabolized folic acid, but not total folate, in plasma has been found to be related to a decrease in natural killer cell cytotoxicity (104). The causative role for unmetabolized folic acid in this study has been questioned (3), and there are still gaps in understanding the factors that contribute to unmetabolized synthetic folic acid accumulation in plasma and the metabolic effects (99).

## Requirement and recommended intakes

The EFSA concluded in 2014 for adults on an average requirement (AR) of 250 μg DFE per day and a population reference intake (PRI) of 330 μg DFE per day. This was based on the folate intake required to maintain folate adequacy characterised by serum folate of ≥ 10 nmol/L and RBC folate concentrations of 340 nmol/L. They assumed a coefficient of variation (CV) of 15% to account for the additional variability associated with the higher requirement for folate in individuals with the MTHFR 677TT genotype (105).

A dietary folate intake of at least 350 µg per day has been considered necessary to prevent an increase in plasma homocysteine levels of the adult population (18). In a study on elderly men and women (66–94 years), a gradual decrease in plasma tHcy from ~11.0 to ~8.5 µmol/L was evident with increasing folate intake ranging from 160 to ~850 µg per day (106). An intake of ~300 µg folate per day was associated with a plasma tHcy of ~10 µmol/L, indicating that a higher folate intake than recommended by NNR2012 (AR for adults: 200 µg/day and RI to 300 µg/day) may improve folate status.

### Women of reproductive age

To promote optimal NTD risk reduction at the population level, the WHO recommends that the RBC folate concentrations should be above a threshold of 906 nmol/L (400 ng/mL) in women of reproductive age (15). It is considered that an RBC folate concentration below this level indicates folate insufficiency and suboptimal NTD prevention. An RBC level of 906 nmol/L corresponds to a plasma/serum folate concentration threshold of 25.5 nmol/L (107). This level coincides with increased genomic stability and stable plasma tHcy concentrations (Figure 2) (108).

As far from all pregnancies are planned, an RI of 400 µg per day for all women of reproductive ages is considered necessary to provide adequate folate status of women experiencing unplanned pregnancies. It has been shown that women may reach a preventive RBC folate concentration of more than 906 nmol/L within 4 weeks of supplementation with daily intake of 800 µg folic acid (17). The prevalence of having a RBC folate <906 nmol/L was 35% after 40 weeks with a daily folic acid supplement of 140 µg and 18% with 400 µg (109). Median folate intake was 227 μg per day for Swedish women of reproductive age (16). Finnish studies report mean folate intakes in women of reproductive ages ranging from 215 to 230 μg per day (65, 66)

### Pregnant women

In NNR2004, the recommended intake during pregnancy was set to 500 µg per day. This was based on previous studies indicating that 400–500 µg per day was considered sufficient to meet the increased requirement from fast-growing foetal tissues during pregnancy (67) and the recommendation was kept unchanged in NNR2012.

The EFSA Panel considers that it is not possible to set an AR for pregnancy and proposed an adequate intake (AI) for folate for pregnancy at 600 μg DFE per day based on a study on maintenance of serum and RBC folate concentrations in pregnancy (105). This study reported that a total of 450 µg per day of dietary folate in addition to synthetic folic acid was sufficient to maintain folate status in pregnant women. This level of intake was considered equivalent to ~600 µg per day dietary equivalents, assuming 50 and 75% availability of dietary folate and synthetic folic acid consumed with meals, respectively (110).

In the Norwegian MoBa study, women who were regular folic acid supplement users had a total mean intake of folate of 615 (SD 270) µg per day of which mean 275 (SD 95) µg per day came from diet alone (111). In pregnancy week 18, women from the MoBa study, with a regular intake of folic acid supplement from 4 weeks before pregancy to week 17, had median serum folate 15.7 (IQR 9.4–23.1) nmol/L compared to median 10.2 (IQR 7.3–16.6) nmol/L in irregular users and median 5.7 (IQR 4.3–7.7) nmol/L in non-users (112), showing that a regular intake of folic acid supplements is necessary to achieve an adequate folate status. However, even among regular supplement users, less than 25% had a serum folate in the range of 25.5 nmol/L, considered optimal for NTD protection. Based on this, recommendations on folate intake should be adjusted. Additionally, folate supplementation should if necessary be combined with vitamin B_12_ supplementation, in view of the commonly found low B_12_ status in pregnancy and the risk of harm to the child of high folate and low B_12_ (113). This recommendation applies especially to women who are vegans and not currently taking a B_12_ supplement (114).

### Lactation

The NNR2012 recommended 500 µg per day to lactating women, and this amount was also considered to allow replenishment of stores before a possible new pregnancy. This was based on the following reasoning: the concentration of folate in human milk varies throughout the lactation period and is highest between 3 and 6 months (115). Smith and co-workers reported the average concentration of folate in human milk to be 85 µg/L (116). Based on a daily milk production of 0.75 L and a bioavailability of 50%, the diet should contain approximately 100 µg of extra folate.

The EFSA added an additional intake of 130 μg DFE per day to the AR for non-lactating women considered to cover folate losses via breast milk, and a PRI of 500 μg DFE per day was derived for lactating women (105).

### Infants and children

The calculated folate intake for infants from birth to 6 months of age was estimated by the EFSA to be 64 μg per day, based on a mean folate concentration of mature breast milk ~80 μg/L (range 45–99 μg/L) (105) and mean breast milk intake per day the first 6 months ~0.8 L per day. In a study based on healthy, well-nourished lactating mothers and infants published in 1980, the mean breast milk folate level was 141.4 μg/L and total daily folate intake for breastfed infants was assessed at 14–25 μg/kg body weight (117). Approximately the same levels were reported in a more recent study from Korea (47). Mean breast milk folate contents ranged from 88.4 to 160.6 μg/L with an overall mean of 128.0 μg/L, and the contents peaked at 2 months postpartum. Folate intake in the infants ranged between 100 and 140 μg per day during the first 12 months. Serum folate in the infants at age 5 months was mean 72 (SD 39) nmol/L and at 12 months mean 76 (SD 40) nmol/L with adequate plasma tHcy levels of mean 4.4 (SD 1.5) and 3.5 (0.8) μmol/L at 5 and 12 months, respectively (47). Mainly breastfed Norwegian infants have high serum folate levels the first 6 months of life [median 31.6 (IQR 21.3–43.3) nmol/L], indicating that folate content in breast milk is adequate (14).

For infants aged 7–11 months, the EFSA recommended an AI of 80 μg DFE per day, by extrapolating upwards from the estimated folate intake in exclusively breastfed infants, considering the metabolically active body mass [median weight or infants at 3 months (6.1 kg) and at 9 months (8.6 kg)] (105).

For older children, the EFSA extrapolated the ARs from the AR for adults using allometric scaling and growth factors and considering differences in reference weights. PRIs ranging from 120 μg DFE per day for age group 1–3 years to 330 μg DFE per day for age group 15–17 years were derived (105).

In a study from Germany, only children who ate food enriched with folic acid had a folate intake corresponding to recommended EFSA intake. For age group 6–12 months, the folate intake was ~105 μg DFE per day and increased to 323 μg DFE per day in age group 15–18 years, compared to infants who did not eat folic acid-enriched food: ranging from ~63 μg DFE per day to 164 μg DFE per day from 6–12 months to 15–18 years (118).

During the first 2 months of life, exclusively breastfed low-birth-weight (<2,500 g) and/or preterm infants (≤32 gestational weeks) could be at risk for folate deficiency, especially when mothers are smokers and/or do not receive folic acid supplementation during pregnancy (119).

## Integration

Recommended intake of folate for various age groups must depend on what is considered an adequate status according to levels of serum folate and the metabolic marker tHcy and not only on the absence of clinical signs of folate deficiency. Based on the recommended clinical decision levels indicating deficency, serum folate needs to be >10 nmol/L in adults and red cell folate >906 nmol/L in women of fertile age.

When estimating folate requirements, one must consider different bioavailability and functionality between synthetic folic acid and dietary folate, along with increased needs of folate in women of fertile age, pregnant and lactating women, preterm and small for gestational age weight infants and individuals who are homozygote for the *MTHFR* gene polymorphism. Adequate levels of both folate and vitamin B_12_ are necessary for an optimal intracellular metabolism, and folate status needs to be reviewed together with serum vitamin B_12_.

## Conflict of interest and funding

The authors have not received any funding or benefits from industry or elsewhere to conduct this study.

## References

[1] Ohrvik VE, Witthoft CM. Human folate bioavailability. Nutrients 2011; 3(4): 475–90. 10.3390/nu3040475 22254106 PMC3257685

[2] Bailey LB, Gregory JF, 3rd. Folate metabolism and requirements. J Nutr 1999; 129(4): 779–82. 10.1093/jn/129.4.779 10203550

[3] Bailey LB, Stover PJ, McNulty H, Fenech MF, Gregory JF, 3rd, Mills JL, et al. Biomarkers of nutrition for development-folate review. J Nutr 2015; 145(7): 1636S–80S. 10.3945/jn.114.206599 26451605 PMC4478945

[4] Kim YI. Folate and cancer: a tale of Dr. Jekyll and Mr. Hyde? Am J Clin Nutr 2018; 107(2): 139–42. 10.1093/ajcn/nqx076 29529163

[5] Li Y, Huang T, Zheng Y, Muka T, Troup J, Hu FB. Folic Acid supplementation and the risk of cardiovascular diseases: a meta-analysis of randomized controlled trials. J Am Heart Assoc 2016; 5(8): e003768. 10.1161/JAHA.116.003768 27528407 PMC5015297

[6] Han YY, Blatter J, Brehm JM, Forno E, Litonjua AA, Celedon JC. Diet and asthma: vitamins and methyl donors. Lancet Respir Med 2013; 1(10): 813–22. 10.1016/S2213-2600(13)70126-7 24461761 PMC3904132

[7] Parr CL, Magnus MC, Karlstad O, Haugen M, Refsum H, Ueland PM, et al. Maternal folate intake during pregnancy and childhood asthma in a population-based cohort. Am J Respir Crit Care Med 2017; 195(2): 221–8. 10.1164/rccm.201604-0788OC 27518161 PMC5394786

[8] Doets EL, Ueland PM, Tell GS, Vollset SE, Nygard OK, Van’t Veer P, et al. Interactions between plasma concentrations of folate and markers of vitamin B(12) status with cognitive performance in elderly people not exposed to folic acid fortification: the Hordaland Health Study. Br J Nutr 2014; 111(6): 1085–95. 10.1017/S000711451300336X 24229560

[9] Malouf R, Grimley Evans J. Folic acid with or without vitamin B12 for the prevention and treatment of healthy elderly and demented people. Cochrane Database Syst Rev. 2008; 4: CD004514. 10.1002/14651858.CD004514.pub2 PMC12926861 18843658

[10] Lassi ZS, Salam RA, Haider BA, Bhutta ZA. Folic acid supplementation during pregnancy for maternal health and pregnancy outcomes. Cochrane Database Syst Rev 2013; 3: CD006896. 10.1002/14651858.CD006896.pub2 PMC10069458 23543547

[11] Scholl TO, Johnson WG. Folic acid: influence on the outcome of pregnancy. Am J Clin Nutr 2000; 71(5 Suppl): 1295S–303S. 10.1093/ajcn/71.5.1295s 10799405

[12] MRC Vitamin Study Research Group. Prevention of neural tube defects: results of the Medical Research Council Vitamin Study. Lancet 1991; 338(8760): 131–7. 10.1016/0140-6736(91)90133-A 1677062

[13] Kancherla V, Botto LD, Rowe LA, Shlobin NA, Caceres A, Arynchyna-Smith A, et al. Preventing birth defects, saving lives, and promoting health equity: an urgent call to action for universal mandatory food fortification with folic acid. Lancet Glob Health 2022; 10(7): e1053–7. 10.1016/S2214-109X(22)00213-3 35617975

[14] Monsen AL, Refsum H, Markestad T, Ueland PM. Cobalamin status and its biochemical markers methylmalonic acid and homocysteine in different age groups from 4 days to 19 years. Clin Chem 2003; 49(12): 2067–75. 10.1373/clinchem.2003.019869 14633879

[15] Cordero AM, Crider KS, Rogers LM, Cannon MJ, Berry RJ. Optimal serum and red blood cell folate concentrations in women of reproductive age for prevention of neural tube defects: World Health Organization guidelines. MMWR Morb Mortal Wkly Rep 2015; 64(15): 421–3. 10.1007/s00394-016-1328-4 25905896 PMC5779552

[16] Ohrvik V, Lemming EW, Nalsen C, Becker W, Ridefelt P, Lindroos AK. Dietary intake and biomarker status of folate in Swedish adults. Eur J Nutr 2018; 57(2): 451–62. 10.1007/s00394-016-1328-4 27787623 PMC5845621

[17] Bramswig S, Prinz-Langenohl R, Lamers Y, Tobolski O, Wintergerst E, Berthold HK, et al. Supplementation with a multivitamin containing 800 microg of folic acid shortens the time to reach the preventive red blood cell folate concentration in healthy women. Int J Vitam Nutr Res 2009; 79(2): 61–70. 10.1024/0300-9831.79.2.61 20108207

[18] De Bree A, Van Dusseldorp M, Brouwer IA, Van het Hof KH, Steegers-Theunissen RP. Folate intake in Europe: recommended, actual and desired intake. Eur J Clin Nutr 1997; 51(10): 643–60. 10.1038/sj.ejcn.1600467 9347284

[19] Blomhoff R, Andersen R, Arnesen EK, Christensen JJ, Eneroth H, Erkkola M, et al. Nordic Nutrition Recommendations 2023. Copenhagen: Nordic Council of Ministers; 2023.

[20] Christensen JJ, Arnesen EK, Andersen R, Eneroth H, Erkkola M, Hoyer A, et al. The Nordic Nutrition Recommendations 2022 – principles and methodologies. Food Nutr Res 2020; 64: 4402. 10.29219/fnr.v64.4402 PMC7307430 32612489

[21] Donovan S, Dewey K, Novotny R, Stang J, Taveras E, Kleinman R, et al. Folic Acid from Fortified Foods and/or Supplements during Pregnancy and Lactation and Health Outcomes: A Systematic Review [Internet]. Alexandria (VA): USDA Nutrition Evidence Systematic Review; 2020 Jul. PMID: . 35289987

[22] Butterworth CE Jr., Baugh CM, Krumdieck C. A study of folate absorption and metabolism in man utilizing carbon-14 – labeled polyglutamates synthesized by the solid phase method. J Clin Invest 1969; 48(6): 1131–42. 10.1172/JCI106070 4977032 PMC322328

[23] Laanpere M, Altmae S, Stavreus-Evers A, Nilsson TK, Yngve A, Salumets A. Folate-mediated one-carbon metabolism and its effect on female fertility and pregnancy viability. Nutr Rev 2010; 68(2): 99–113. 10.1111/j.1753-4887.2009.00266.x 20137055

[24] Bhandari SD, Gregory JF, 3rd. Folic acid, 5-methyl-tetrahydrofolate and 5-formyl-tetrahydrofolate exhibit equivalent intestinal absorption, metabolism and in vivo kinetics in rats. J Nutr 1992; 122(9): 1847–54. 10.1093/jn/122.9.1847 1512634

[25] Gregory JF, 3rd. Bioavailability of folate. Eur J Clin Nutr 1997; 51(Suppl 1): S54–9. 9023482

[26] Brouwer IA, Van Dusseldorp M, West CE, Meyboom S, Thomas CM, Duran M, et al. Dietary folate from vegetables and citrus fruit decreases plasma homocysteine concentrations in humans in a dietary controlled trial. J Nutr 1999; 129(6): 1135–9. 10.1093/jn/129.6.1135 10356077

[27] Hannon-Fletcher MP, Armstrong NC, Scott JM, Pentieva K, Bradbury I, Ward M, et al. Determining bioavailability of food folates in a controlled intervention study. Am J Clin Nutr 2004; 80(4): 911–8. 10.1093/ajcn/80.4.911 15447898

[28] Bjork M, Riedel B, Spigset O, Veiby G, Kolstad E, Daltveit AK, et al. Association of folic acid supplementation during pregnancy with the risk of autistic traits in children exposed to antiepileptic drugs in utero. JAMA Neurol 2018; 75(2): 160–8. 10.1001/jamaneurol.2017.3897 29279889 PMC5838632

[29] Clark SL. Oral folic acid tolerance test in normal human subjects and patients with pernicious anemia. Proc Soc Exp Biol Med Soc Exp Biol Med 1958; 82: 25–7. 10.3181/00379727-82-20011 13037790

[30] Dietary reference intakes for thiamin, riboflavin, niacin, vitamin B6, folate, vitamin B12, pantothenic acid, biotin, and choline. Washington, DC: The National Academies Collection: Reports funded by National Institutes of Health; 1998. 23193625

[31] Jacques PF, Bostom AG, Williams RR, Ellison RC, Eckfeldt JH, Rosenberg IH, et al. Relation between folate status, a common mutation in methylenetetrahydrofolate reductase, and plasma homocysteine concentrations. Circulation 1996; 93: 7–9. 10.1161/01.CIR.93.1.7 8616944

[32] Huang X, Qin X, Yang W, Liu L, Jiang C, Zhang X, et al. MTHFR gene and serum folate interaction on serum homocysteine lowering: prospect for precision folic acid treatment. Arteriosc Thromb Vascu Biol 2018; 38(3): 679–85. 10.1161/ATVBAHA.117.310211 29371246

[33] Hibbard BM. The role of folic acid in pregnancy with particular reference to anaemia, abruption and abortion. J Obstet Gynaecol Br Commonw 1964; 71: 529–42. 10.1111/j.1471-0528.1964.tb04317.x 14194440

[34] McPartlin J, Halligan A, Scott JM, Darling M, Weir DG. Accelerated folate breakdown in pregnancy. Lancet 1993; 341(8838): 148–9. 10.1016/0140-6736(93)90007-4 8093747

[35] Rosenblatt DS, Whitehead VM. Cobalamin and folate deficiency: acquired and hereditary disorders in children. Semin Hematol 1999; 36(1): 19–34. 9930566

[36] Allen LH. B vitamins in breast milk: relative importance of maternal status and intake, and effects on infant status and function. Adv Nutr 2012; 3(3): 362–9. 10.3945/an.111.001172 22585913 PMC3649471

[37] Cooperman JM, Dweck HS, Newman LJ, Garbarino C, Lopez R. The folate in human milk. Am J Clin Nutr 1982; 36(4): 576–80. 10.1093/ajcn/36.4.576 6896957

[38] Mackey AD, Picciano MF. Maternal folate status during extended lactation and the effect of supplemental folic acid. Am J Clin Nutr 1999; 69(2): 285–92. 10.1093/ajcn/69.2.285 9989694

[39] Houghton LA, Yang J, O’Connor DL. Unmetabolized folic acid and total folate concentrations in breast milk are unaffected by low-dose folate supplements. Am J Clin Nutr 2009; 89(1): 216–20. 10.3945/ajcn.2008.26564 19056550

[40] Molloy AM, Mills JL, McPartlin J, Kirke PN, Scott JM, Daly S. Maternal and fetal plasma homocysteine concentrations at birth: the influence of folate, vitamin B12, and the 5,10-methylenetetrahydrofolate reductase 677C-->T variant. Am J Obstet Gynecol 2002; 186(3): 499–503. 10.1067/mob.2002.121105 11904614

[41] Baker H, Thind IS, Frank O, DeAngelis B, Caterini H, Louria DB. Vitamin levels in low-birth-weight newborn infants and their mothers. Am J Obstet Gynecol 1977; 129(5): 521–4. 10.1016/0002-9378(77)90090-4 910841

[42] Pathak A, Godwin HA. Vitamin B 12 and folic acid values in premature infants. Pediatrics 1972; 50(4): 584–9. 10.1542/peds.50.4.584 5073009

[43] Samuel PD, Burland WL, Simpson K. Response to oral administration of pteroylmonoglutamic acid or pteroylpolyglutamate in newborn infants of low birth weight. Br J Nutr 1973; 30(2): 165–9. 10.1079/BJN19730021 4800368

[44] Bjorke-Monsen AL, Ueland PM. Cobalamin status in children. J Inherit Metab Dis 2011; 34(1): 111–9. 10.1007/s10545-010-9119-1 20508991

[45] Montgomery JA, Clayton SJ, Thomas HJ, Shannon WM, Arnett G, Bodner AJ, et al. Carbocyclic analogue of 3-deazaadenosine. A novel antiviral agent using S-adenosylhomocysteine hydrolase as a pharmacological target. J Med Chem 1982; 25: 626–9. 10.1021/jm00348a004 7097716

[46] Salmenpera L, Perheentupa J, Siimes MA. Folate nutrition is optimal in exclusively breast-fed infants but inadequate in some of their mothers and in formula-fed infants. J Pediatr Gastroenterol Nutr 1986; 5(2): 283–9. 10.1097/00005176-198605020-00021 3958855

[47] Han YH, Yon M, Han HS, Kim KY, Tamura T, Hyun TH. Folate contents in human milk and casein-based and soya-based formulas, and folate status in Korean infants. Br J Nutr 2009; 101(12): 1769–74. 10.1017/S0007114508158974 19079945

[48] Howard MR, Turnbull AJ, Morley P, Hollier P, Webb R, Clarke A. A prospective study of the prevalence of undiagnosed coeliac disease in laboratory defined iron and folate deficiency. J Clin Pathol 2002; 55(10): 754–7. 10.1136/jcp.55.10.754 12354801 PMC1769776

[49] Mitchell ES, Snell ES, Williams RJ. Folate. In: Combs GF, ed. The vitamins fundamental aspects in nutrition and health. North Dakota: Academic Press; 1998, pp. 377–401.

[50] Hunt SE, Netting MJ, Sullivan TR, Best KP, Houghton LA, Makrides M, et al. Red Blood cell folate likely overestimated in Australian National Survey: implications for neural tube defect risk. Nutrients 2020; 12(5): 1283. 10.3390/nu12051283 32369938 PMC7281964

[51] Clifford AJ, Noceti EM, Block-Joy A, Block T, Block G. Erythrocyte folate and its response to folic acid supplementation is assay dependent in women. J Nutr 2005; 135(1): 137–43. 10.1093/jn/135.1.137 15623845

[52] Gunter EW, Bowman BA, Caudill SP, Twite DB, Adams MJ, Sampson EJ. Results of an international round robin for serum and whole-blood folate. Clin Chem 1996; 42(10): 1689–94. 10.1093/clinchem/42.10.1689 8855155

[53] Wright AJ, Finglas PM, Southon S. Erythrocyte folate analysis: a cause for concern? Clin Chem 1998; 44(9): 1886–91. 10.1093/clinchem/44.9.1886 9732972

[54] Wickramasinghe SN. Nutritional anaemias. Clin Lab Haematol 1988; 10: 117–34. 10.1111/j.1365-2257.1988.tb01164.x 3046831

[55] Bull CF, Mayrhofer G, Zeegers D, Mun GL, Hande MP, Fenech MF. Folate deficiency is associated with the formation of complex nuclear anomalies in the cytokinesis-block micronucleus cytome assay. Environ Mol Mutagen 2012; 53(4): 311–23. 10.1002/em.21688 22430981

[56] Bjorke-Monsen AL. Hva betyr en høy plasma-homocysteinverdi? [What does a high plasma homocysteine level signify?]. Tidsskr Nor Laegeforen. 2021 Mar 22;141(5). 10.4045/tidsskr.21.0023. 33754668

[57] Ozarda Y, Sikaris K, Streichert T, Macri J, Intervals ICoR, Decision L. Distinguishing reference intervals and clinical decision limits – A review by the IFCC Committee on Reference Intervals and Decision Limits. Crit Rev Clin Lab Sci 2018; 55(6): 420–31. 10.1080/10408363.2018.1482256 30047297

[58] Steluti J, Selhub J, Paul L, Reginaldo C, Fisberg RM, Marchioni DML. An overview of folate status in a population-based study from Sao Paulo, Brazil and the potential impact of 10 years of national folic acid fortification policy. Eur J Clin Nutr 2017; 71(10): 1173–8. 10.1038/ejcn.2017.60 28488686

[59] WHO. [cited 24 June 2022]. Available from: https://www.who.int/elena/titles/guidance_summaries/daily_iron_pregnancy/en/

[60] Bjorke-Monsen AL, Renstrom R. What is optimal folate status? Tidsskr Nor Laegeforen. 2020 May 4;140(7). 10.4045/tidsskr.19.0588. PMID: . 32378856

[61] Bjørke-Monsen AL, Roth C, Magnus P, Midttun Ø, Nilsen RM, Reichborn-Kjennerud T, et al. Maternal B vitamin status in pregnancy week 18 according to reported use of folic acid supplements. Mol Nutr Food Res 2013; 57(4): 645–52. 10.1002/mnfr.201200114 23001761 PMC3774931

[62] Caudill MA. Folate bioavailability: implications for establishing dietary recommendations and optimizing status. Am J Clin Nutr 2010; 91(5): 1455S–60S. 10.3945/ajcn.2010.28674E 20219964 PMC2854911

[63] Lemming EW, Pitsi T. The Nordic Nutrition Recommendations 2022 – food consumption and nutrient intake in the adult population of the Nordic and Baltic countries. Food Nutr Res 2022; 66: 8572. 10.29219/fnr.v66.8572 PMC9199833 35757440

[64] Medici V, Halsted CH. Folate, alcohol, and liver disease. Mol Nutri Food Res 2013; 57(4): 596–606. 10.1002/mnfr.201200077 PMC3736728 23136133

[65] Moazzen S, Dolatkhah R, Tabrizi JS, Shaarbafi J, Alizadeh BZ, de Bock GH, et al. Folic acid intake and folate status and colorectal cancer risk: a systematic review and meta-analysis. Clin Nutr 2018; 37(6 Pt A): 1926–34. 10.1016/j.clnu.2017.10.010 29132834

[66] Zhang D, Wen X, Wu W, Guo Y, Cui W. Elevated homocysteine level and folate deficiency associated with increased overall risk of carcinogenesis: meta-analysis of 83 case-control studies involving 35,758 individuals. PLoS One 2015; 10(5): e0123423. 10.1371/journal.pone.0123423 25985325 PMC4436268

[67] Pieroth R, Paver S, Day S, Lammersfeld C. Folate and its impact on cancer risk. Curr Nutr Rep 2018; 7(3): 70–84. 10.1007/s13668-018-0237-y 30099693 PMC6132377

[68] Bird CL, Swendseid ME, Witte JS, Shikany JM, Hunt IF, Frankl HD, et al. Red cell and plasma folate, folate consumption, and the risk of colorectal adenomatous polyps. Cancer Epidemiol Biomarkers Prev 1995; 4(7): 709–14. 8672986

[69] Gao QY, Chen HM, Chen YX, Wang YC, Wang ZH, Tang JT, et al. Folic acid prevents the initial occurrence of sporadic colorectal adenoma in Chinese older than 50 years of age: a randomized clinical trial. Cancer Prev Res (Phila) 2013; 6(7): 744–52. 10.1158/1940-6207.CAPR-13-0013 23682073

[70] Gibson TM, Weinstein SJ, Pfeiffer RM, Hollenbeck AR, Subar AF, Schatzkin A, et al. Pre- and postfortification intake of folate and risk of colorectal cancer in a large prospective cohort study in the United States. Am J Clin Nutr 2011; 94(4): 1053–62. 10.3945/ajcn.110.002659 21813806 PMC3173023

[71] Konings EJ, Goldbohm RA, Brants HA, Saris WH, Van den Brandt PA. Intake of dietary folate vitamers and risk of colorectal carcinoma: results from The Netherlands Cohort Study. Cancer 2002; 95(7): 1421–33. 10.1002/cncr.10866 12237910

[72] Fu H, He J, Li C, Deng Z, Chang H. Folate intake and risk of colorectal cancer: a systematic review and up-to-date meta-analysis of prospective studies. Eur J Cancer Prev 2022; 32(2): 103–12. 10.1097/CEJ.0000000000000744 35579178

[73] Smith AD, Refsum H. Homocysteine – from disease biomarker to disease prevention. J Intern Med 2021; 290(4): 826–54. 10.1111/joim.13279 33660358

[74] Park JH, Saposnik G, Ovbiagele B, Markovic D, Towfighi A. Effect of B-vitamins on stroke risk among individuals with vascular disease who are not on antiplatelets: a meta-analysis. Int J Stroke 2016; 11(2): 206–11. 10.1177/1747493015616512 26783312

[75] Huo Y, Li J, Qin X, Huang Y, Wang X, Gottesman RF, et al. Efficacy of folic acid therapy in primary prevention of stroke among adults with hypertension in China: the CSPPT randomized clinical trial. JAMA 2015; 313(13): 1325–35. 10.1001/jama.2015.2274 25771069

[76] Nilsson TK, Yngve A, Bottiger AK, Hurtig-Wennlof A, Sjostrom M. High folate intake is related to better academic achievement in Swedish adolescents. Pediatrics 2011; 128(2): e358–65. 10.1542/peds.2010-1481 21746721

[77] Nurk E, Refsum H, Tell GS, Engedal K, Vollset SE, Ueland PM, et al. Plasma total homocysteine and memory in the elderly: the Hordaland Homocysteine Study. Ann Neurol 2005; 58(6): 847–57. 10.1002/ana.20645 16254972

[78] Wang Z, Zhu W, Xing Y, Jia J, Tang Y. B vitamins and prevention of cognitive decline and incident dementia: a systematic review and meta-analysis. Nutr Rev 2022; 80(4): 931–49. 10.1093/nutrit/nuab057 34432056

[79] Ramos MI, Allen LH, Mungas DM, Jagust WJ, Haan MN, Green R, et al. Low folate status is associated with impaired cognitive function and dementia in the Sacramento Area Latino Study on Aging. Am J Clin Nutr 2005; 82(6): 1346–52. 10.1093/ajcn/82.6.1346 16332669

[80] Swinburn BA, Sacks G, Hall KD, McPherson K, Finegood DT, Moodie ML, et al. The global obesity pandemic: shaped by global drivers and local environments. Lancet 2011; 378(9793): 804–14. 10.1016/S0140-6736(11)60813-1 21872749

[81] Magnusson M, Sorensen TI, Olafsdottir S, Lehtinen-Jacks S, Holmen TL, Heitmann BL, et al. Social inequalities in obesity persist in the Nordic Region despite its relative affluence and equity. Curr Obes Rep 2014; 3: 1–15. 10.1007/s13679-013-0087-2 24533235 PMC3920028

[82] Kreusler P, Vogel M, Willenberg A, Baber R, Dietz Y, Korner A, et al. Folate and cobalamin serum levels in healthy children and adolescents and their association with age, sex, BMI and socioeconomic status. Nutrients 2021; 13(2): 546. 10.3390/nu13020546 33562369 PMC7915137

[83] Mojtabai R. Body mass index and serum folate in childbearing age women. Eur J Epidemiol 2004; 19(11): 1029–36. 10.1007/s10654-004-2253-z 15648596

[84] Bjørke-Monsen AL, Ulvik A, Nilsen RM, Midttun Ø, Roth C, Magnus P, et al. Impact of pre-pregnancy BMI on B vitamin and inflammatory status in early pregnancy: an observational cohort study. Nutrients 2016; 8(12): 776. 10.3390/nu8120776 27916904 PMC5188431

[85] Kimmons JE, Blanck HM, Tohill BC, Zhang J, Khan LK. Associations between body mass index and the prevalence of low micronutrient levels among US adults. MedGenMed Medscape Gen Med 2006; 8(4): 59. PMC1868363 17415336

[86] De-Regil LM, Pena-Rosas JP, Fernandez-Gaxiola AC, Rayco-Solon P. Effects and safety of periconceptional oral folate supplementation for preventing birth defects. Cochrane Database Syst Rev 2015; 12: CD007950. 10.1002/14651858.CD007950.pub3 PMC8783750 26662928

[87] Viswanathan M, Treiman KA, Kish-Doto J, Middleton JC, Coker-Schwimmer EJ, Nicholson WK. Folic acid supplementation for the prevention of neural tube defects: an updated evidence report and systematic review for the US preventive services task force. JAMA 2017; 317(2): 190–203. 10.1001/jama.2016.19193 28097361

[88] Greenberg JA, Bell SJ, Guan Y, Yu YH. Folic Acid supplementation and pregnancy: more than just neural tube defect prevention. Rev Obstetr Gynecol 2011; 4(2): 52–9. PMC3218540 22102928

[89] Castro K, Klein Lda S, Baronio D, Gottfried C, Riesgo R, Perry IS. Folic acid and autism: what do we know? Nutr Neurosci 2016; 19(7): 310–7. 10.1179/1476830514Y.0000000142 25087906

[90] Gao Y, Sheng C, Xie RH, Sun W, Asztalos E, Moddemann D, et al. New perspective on impact of folic acid supplementation during pregnancy on neurodevelopment/autism in the offspring children – A systematic review. PLoS One 2016; 11(11): e0165626. 10.1371/journal.pone.0165626 27875541 PMC5119728

[91] Caffrey A, McNulty H, Rollins M, Prasad G, Gaur P, Talcott JB, et al. Effects of maternal folic acid supplementation during the second and third trimesters of pregnancy on neurocognitive development in the child: an 11-year follow-up from a randomised controlled trial. BMC Med 2021; 19(1): 73. 10.1186/s12916-021-01914-9 33750355 PMC7945668

[92] Julvez J, Fortuny J, Mendez M, Torrent M, Ribas-Fito N, Sunyer J. Maternal use of folic acid supplements during pregnancy and four-year-old neurodevelopment in a population-based birth cohort. Paediatr Perinat Epidemiol 2009; 23(3): 199–206. 10.1111/j.1365-3016.2009.01032.x 19775381

[93] Roza SJ, Van Batenburg-Eddes T, Steegers EA, Jaddoe VW, Mackenbach JP, Hofman A, et al. Maternal folic acid supplement use in early pregnancy and child behavioural problems: the Generation R Study. Br J Nutr 2010; 103(3): 445–52. 10.1017/S0007114509991954 19772683

[94] Valera-Gran D, Garcia de la Hera M, Navarrete-Munoz EM, Fernandez-Somoano A, Tardon A, Julvez J, et al. Folic acid supplements during pregnancy and child psychomotor development after the first year of life. JAMA Pediatr 2014; 168(11): e142611. 10.1001/jamapediatrics.2014.2611 25365251

[95] Brown SB, Reeves KW, Bertone-Johnson ER. Maternal folate exposure in pregnancy and childhood asthma and allergy: a systematic review. Nutr Rev 2014; 72(1): 55–64. 10.1111/nure.12080 24551950

[96] Hua X, Zhang J, Guo Y, Shen M, Gaudet L, Janoudi G, et al. Effect of folic acid supplementation during pregnancy on gestational hypertension/preeclampsia: a systematic review and meta-analysis. Hypertens Pregnan 2016; 35(4): 447–60. 10.1080/10641955.2016.1183673 27315401

[97] Yajnik CS, Deshpande SS, Jackson AA, Refsum H, Rao S, Fisher DJ, et al. Vitamin B12 and folate concentrations during pregnancy and insulin resistance in the offspring: the Pune Maternal Nutrition Study. Diabetologia 2008; 51(1): 29–38. 10.1007/s00125-007-0793-y 17851649 PMC2100429

[98] Scientific Committee on Food (SCF) and the EFSA Panel on Dietetic Products NaAN. Overview on Tolerable Upper Intake Levels as derived by the Scientific Committee on Food (SCF) and the EFSA Panel on Dietetic Products, Nutrition and Allergies (NDA) Summary of Tolerable Upper Intake Levels – version 4. 2018. https://www.efsa.europa.eu/sites/default/files/assets/UL_Summary_tables.pdf

[99] Maruvada P, Stover PJ, Mason JB, Bailey RL, Davis CD, Field MS, et al. Knowledge gaps in understanding the metabolic and clinical effects of excess folates/folic acid: a summary, and perspectives, from an NIH workshop. Am J Clin Nutr 2020; 112(5): 1390–403. 10.1093/ajcn/nqaa259 33022704 PMC7657327

[100] Selhub J, Miller JW, Troen AM, Mason JB, Jacques PF. Perspective: the high-folate-low-vitamin B-12 interaction is a novel cause of vitamin B-12 depletion with a specific etiology-a hypothesis. Adv Nutr 2022; 13(1): 16–33. 10.1093/advances/nmab106 34634124 PMC8803489

[101] Butterworth CE Jr, Tamura T. Folic acid safety and toxicity: a brief review. Am J Clin Nutr 1989; 50(2): 353–8. 10.1093/ajcn/50.2.353 2667316

[102] Pfeiffer CM, Sternberg MR, Fazili Z, Yetley EA, Lacher DA, Bailey RL, et al. Unmetabolized folic acid is detected in nearly all serum samples from US children, adolescents, and adults. J Nutr 2015; 145(3): 520–31. 10.3945/jn.114.201210 25733468 PMC4336532

[103] Morris MS, Jacques PF, Rosenberg IH, Selhub J. Circulating unmetabolized folic acid and 5-methyltetrahydrofolate in relation to anemia, macrocytosis, and cognitive test performance in American seniors. Am J Clin Nutr 2010; 91(6): 1733–44. 10.3945/ajcn.2009.28671 20357042

[104] Troen AM, Mitchell B, Sorensen B, Wener MH, Johnston A, Wood B, et al. Unmetabolized folic acid in plasma is associated with reduced natural killer cell cytotoxicity among postmenopausal women. J Nutr 2006; 136(1): 189–94. 10.1093/jn/136.1.189 16365081

[105] EFSA Panel on Dietetic Products NaAN. SCIENTIFIC OPINION Scientific Opinion on Dietary Reference Values for folate. EFSA J. 2014; 12(11): 3893. 10.2903/j.efsa.2014.3893 PMC13136993 42087961

[106] Koehler KM, Baumgartner RN, Garry PJ, Allen RH, Stabler SP, Rimm EB. Association of folate intake and serum homocysteine in elderly persons according to vitamin supplementation and alcohol use. Am J Clin Nutr 2001; 73(3): 628–37. 10.1093/ajcn/73.3.628 11237942

[107] Chen MY, Rose CE, Qi YP, Williams JL, Yeung LF, Berry RJ, et al. Defining the plasma folate concentration associated with the red blood cell folate concentration threshold for optimal neural tube defects prevention: a population-based, randomized trial of folic acid supplementation. Am J Clin Nutr 2019; 109(5): 1452–61. 10.1093/ajcn/nqz027 31005964 PMC7099800

[108] Fenech M. The role of folic acid and Vitamin B12 in genomic stability of human cells. Mutat Res 2001; 475(1–2): 57–67. 10.1016/S0027-5107(01)00079-3 11295154

[109] Hursthouse NA, Gray AR, Miller JC, Rose MC, Houghton LA. Folate status of reproductive age women and neural tube defect risk: the effect of long-term folic acid supplementation at doses of 140 microg and 400 microg per day. Nutrients 2011; 3(1): 49–62. 10.3390/nu3010049 22254076 PMC3257734

[110] Caudill MA, Cruz AC, Gregory JF, 3rd, Hutson AD, Bailey LB. Folate status response to controlled folate intake in pregnant women. J Nutr 1997; 127(12): 2363–70. 10.1093/jn/127.12.2363 9405587

[111] Haugen M, Brantsaeter AL, Alexander J, Meltzer HM. Dietary supplements contribute substantially to the total nutrient intake in pregnant Norwegian women. Ann Nutr Metab 2008; 52(4): 272–80. 10.1159/000146274 18645244 PMC2813797

[112] Bjorke-Monsen AL, Roth C, Magnus P, Midttun O, Nilsen RM, Reichborn-Kjennerud T, et al. Maternal B vitamin status in pregnancy week 18 according to reported use of folic acid supplements. Mol Nutr Food Res 2013; 57(4): 645–52. 10.1002/mnfr.201200114 23001761 PMC3774931

[113] Green R, Allen LH, Bjorke-Monsen AL, Brito A, Gueant JL, Miller JW, et al. Vitamin B12 deficiency. Nat Rev Dis Prim 2017; 3: 17040. 10.1038/nrdp.2017.40 28660890

[114] Pawlak R. To vegan or not to vegan when pregnant, lactating or feeding young children. Eur J Clin Nutr 2017; 71(11): 1259–62. 10.1038/ejcn.2017.111 28745335

[115] Smith AM, Picciano MF, Deering RH. Folate intake and blood concentrations of term infants. Am J Clin Nutr 1985; 41(3): 590–8. 10.1093/ajcn/41.3.590 3976558

[116] Asfour R, Wahbeh N, Waslien CI, Guindi S, Darby WJ. Folacin requirement of children. III. Normal infants. Am J Clin Nutr 1977; 30(7): 1098–105. 10.1093/ajcn/30.7.1098 577655

[117] Tamura T, Yoshimura Y, Arakawa T. Human milk folate and folate status in lactating mothers and their infants. Am J Clin Nutr 1980; 33(2): 193–7. 10.1093/ajcn/33.2.193 7355792

[118] Sichert-Hellert W, Kersting M. Fortifying food with folic acid improves folate intake in German infants, children, and adolescents. J Nutr 2004; 134(10): 2685–90. 10.1093/jn/134.10.2685 15465767

[119] Oncel MY, Calisici E, Ozdemir R, Yurttutan S, Erdeve O, Karahan S, et al. Is folic acid supplementation really necessary in preterm infants </= 32 weeks of gestation? J Pediatr Gastroenterol Nutr 2014; 58(2): 188–92. 10.1097/MPG.0000000000000181 24051483

